# Development of Palatal Growth Charts for Unilateral Cleft Lip and Palate Before Primary Single-Stage Repair

**DOI:** 10.1177/10556656251369672

**Published:** 2025-08-26

**Authors:** Adelita Sommacal, Benito K. Benitez, Yoriko Lill, Maria Fernandez-Pose, Michel Beyer, Florian M. Thieringer, Andreas A. Mueller

**Affiliations:** 1 Oral and Craniomaxillofacial Surgery, 30262 University and University Hospital Basel, Basel, Switzerland; 2 Pediatric Oral and Craniomaxillofacial Surgery, University Children's Hospital Basel, Basel, Switzerland; 3 Department of Biomedical Engineering, 27209 University of Basel, Allschwil, Switzerland; 4 Department of Clinical Research, 27209 University of Basel, Basel, Switzerland

**Keywords:** cleft lip and palate, craniofacial malformation, orofacial cleft, 3D morphology, growth chart

## Abstract

**Objective:** We study changes in palatal morphology under presurgical passive plate therapy (PSPPT) in patients with unilateral cleft lip and palate (UCLP) from birth to single-stage cleft lip and palate repair. The primary aim was to model a growth chart for palatal surface area, in order to generate a reference for individual growth prediction and surgical planning. The secondary aim was to quantify and evaluate morphological changes in the palate using measurements on 3-dimensional (3D) intraoral surface models. **Design:** Retrospective cohort study. **Setting:** Tertiary care university hospital. **Patients:** We analyzed 3D models from intraoral scans and digitized plaster casts of 30 patients with UCLP undergoing PSPPT in their first year of life. **Main Outcome Measures:** We measured areas (palatal surface, projected palatal size, greater and lesser segments), angles (premaxillary/lesser segment rotation), transversal distances (intercanine, intertuberosity, premaxilla, cleft width), and maxillary sagittal length, using 3D software and analyzed the data with linear mixed-effects and nonlinear models. **Results:** The palatal width measured between the canines and tuberosities was unaffected by age, whereas the palatal surface area, premaxillary width and maxillary length increased with age. The premaxilla became more centered, and the anterior cleft width decreased consistently during treatment, despite a notable variation in the starting levels. **Conclusions:** Despite significant variations in baseline palatal size and morphology, the growth rate across all dimensions was consistent among individuals. This suggests that individual palatal growth morphology can be inferred from growth charts, allowing monitoring and prediction of cleft palate development for subsequent surgical planning and timing.

## Introduction

The anatomy of cleft lip and palate (CLP) differs between individuals and between regions of the palate in 3-dimensional (3D) space and topology, with varying degrees of tissue displacement, deformity, and deficit. CLP is a complex congenital anomaly that presents both anatomical and functional rehabilitation challenges. To fully understand this complex malformation, it must be analyzed in 3D, allowing for further delineation of how palatal morphology evolves over time and in response to presurgical therapy. Among evolving treatment strategies, presurgical passive plate therapy (PSPPT) has become an integral part of many cleft centers for infants with unilateral cleft lip and palate (UCLP).^1-4^ Various surgical techniques have been used to treat UCLP, each with its own merits and challenges.^5-10^ Despite advances in treatment that have improved morphological outcomes, the interaction between intrinsic developmental patterns and external modulating factors, such as PSPPT,^11-13^ presurgical orthopedics, surgery, and functional restoration, remains unclear. This uncertainty arises because only the cumulative effect on growth and development can be observed in each patient. In many centers, PSPPT is routinely used to optimize the treatment of UCLP.^1-3^ The goals of PSPPT can be both functional—separating the oral from the nasal cavity, aiming to facilitate feeding and breathing—and structural, helping to narrow the cleft by keeping the tongue out of the cleft palate.^3^

Advances in data science enable the detailed analysis of individual health data, which is particularly valuable for conditions such as pediatric maxillofacial malformations, where growth and treatment are closely linked and influence each other. Precise monitoring, such as tracking weight and length, is essential for personalized treatment. Given the rarity and heterogeneity of cleft conditions, reference data and models are essential to objectively assess their severity, predict individual development, and evaluate treatment outcomes. With advances in digital technologies, many studies have used 3D data to better understand the impact of therapies on palatal morphology.^1,2,8,9,11^ Longitudinal studies with regular patient follow-up allow for a more precise assessment of the impact of treatment on development, which is critical in pediatric care. Growth charts for height and weight are routinely used since decades to monitor children's development during the neonatal and postnatal periods.^14-18^ However, there are only limited number of studies on growth for the healthy palate^19^ and for UCLP.^20^ Growth charts specific to CLP morphology, either for spontaneous growth or for growth exclusively under presurgical therapy, do not yet exist.

This study aimed to develop a growth chart for a cohort of patients with UCLP by analyzing changes in palatal morphological undergoing PSPPT. Intraoral impressions were analyzed from patients with UCLP, planned for single-stage cleft lip and palate repair at around 10 months of age. The study sought to map the growth trajectory and assess the impact of treatment on palatal surface area, providing insights into both the patterns and variability of palatal cleft morphology changes during the first year of life. This contributes to the creation of reference growth charts for UCLP under PSPPT and fills a gap in understanding growth trajectories before surgical intervention.

## Methods

### Study Design, Setting, and Participants

The study sample included patients with non-syndromic unilateral cleft lip and palate. All patients were treated at the University Center for Cleft Lip and Palate and Facial Malformations of the University Hospital Basel between 2000 and 2024. They underwent PSPPT and single-stage cleft lip and palate repair between 4 and 10 months of age.^1,21^

All patients who underwent PSPPT and had intraoral scans or impressions from at least 2 time points were included in this study. This retrospective cohort study was conducted in accordance with the Declaration of Helsinki and approved by the Ethics Committee of Northwestern and Central Switzerland (EKNZ) (BASEC ID: 2020-00388). All parents and guardians provided written informed consent for the surgical procedures and the release of medical information and photographs for scientific purposes. The data analyzed in the study was anonymized and stored in the hospital database to ensure confidentiality.

### Data Sources and Measurements

**Figure 1.**
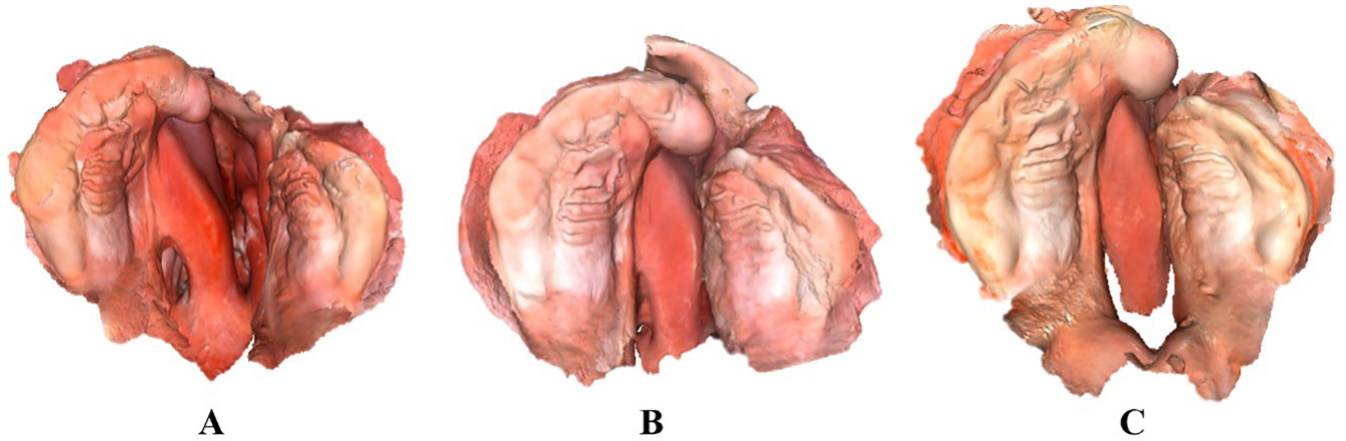
Example of Intraoral Scans of a Patient With Unilateral Cleft lip and Palate at (A) Birth, (B) 3 Months old, and (C) 10 Months old Undergoing Presurgical Passive Plate Therapy.

The 3D morphology of the cleft palate was recorded using either conventional plaster casts or intraoral scans at a minimum of 2 time points, immediately after birth and immediately prior to single-stage cleft lip and palate repair between 4 and 10 months of age. In many cases, additional documentation time points were available due to the need for plate renewal as growth progressed. The 3D data were stored as standard tessellation language (STL) files or object files (OBJ). Previous studies have reported negligible discrepancies between the different methods used to acquire these images.^22^ Patient characteristics, such as age at the time of impression acquisition and birth weight were also recorded. Figure 1 Shows an example of intraoral scans taken with an optical scanner (Medit i500; Medit Corp., Seoul, Korea) at 3 different time points during PSPPT. The workflow detailing scanner selection and scanning procedure in these patients is described in previous publications accompanied with video instructions.^23,24^

**Figure 2.**
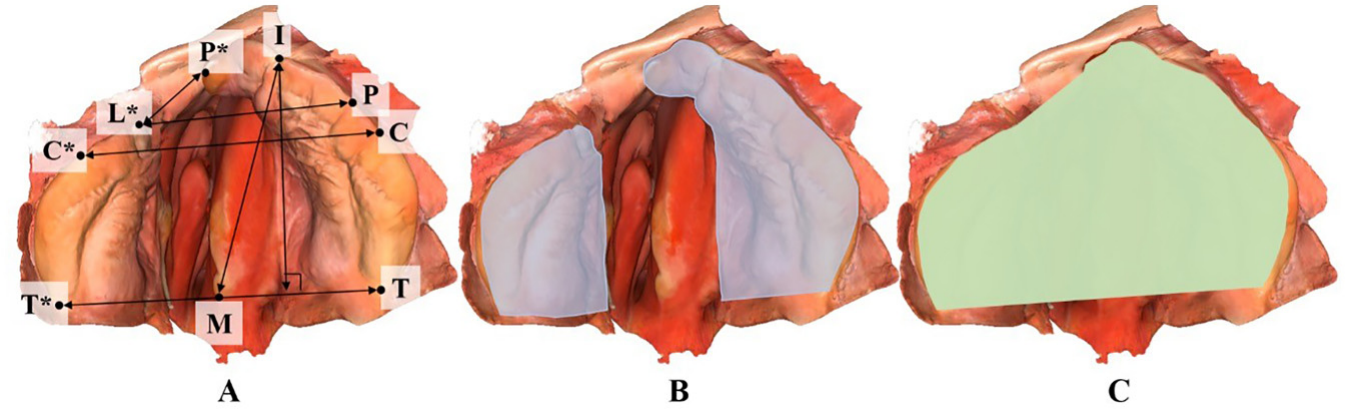
Three-Dimensional (3D) Surface Scans With Landmarks, Measured Distances, Angles, and Measured Areas. Intraoral Scans at 3 Months of age. (A) Definitions of the Landmarks and Distances as Described in Tables 1 and 2. (B) Palatal Mucosa Surface Areas Labeled in Blue. (C) Flat Surface Enclosed by the Alveolar Ridge Projected Onto the Occlusal Plane Labeled in Green. The Posterior Border^1,21^ and the Construction of the Occlusal Plane^19^ are Defined as Described in Previous Studies.

The 3D scans were imported into the 3-matic software (V 17.0, Materialise NV, Leuven, Belgium), where anatomical landmarks were placed on the model manually, except for the intertuberosity midpoint (M) which was automatically determined within 3-matic. The landmarks and their corresponding descriptions are listed in Table 1. The same landmarks described by Benitez et al^1,25^ were used, with adaptations based on previous studies.^8,19,21,26-31^ The distances and angles between specific landmarks were calculated. These measurements, along with their descriptions, are listed in Table 2 and illustrated in Figure 2A. The palatal size was calculated automatically on a 2-dimensional (2D) projection of the manually placed contour. Additionally, the palatal surface area and the surface areas of the left and right alveolar segments as well as the premaxilla were measured in 3D on the manually placed contour following the curvature of the scan/mesh to assess the tissue available for repair (Table 2 and Figure 2B and C).

**Table 1.** Landmarks Used for Measurements Defined in Benitez et al.^1,25^ and Adapted From Previous Studies.^8,19,21,26-31^

Landmarks	Description
I	Interincisive point; landmark in the middle of the premaxilla located on the tip of the ridge on the line between the labial frenulum and the incisive papilla
P, P*	Premaxilla points; most distal landmark on the edge of the premaxilla in the continuation of the alveolar ridge in the sulcus between the deciduous lateral incisor and canine/*most anterior landmark of the greater alveolar segment on the edge of the premaxilla
L*	Lesser segment point; most anterior landmark on the edge of the lesser alveolar segment
C, C*	Canine points; distal margin of canine bulge on the greater/*lesser alveolar segment
T, T*	Tuberosity points; most distal landmark on the greater/*lesser alveolar segment
M	Intertuberosity midpoint; on theline connecting the tuberosity points

**Table 2.** List of the Distances, Angles, and Areas (Adapted From Previous Studies^19,30^).

Distance/angle/area	Definition	Description
C*-C	Intercanine distance	Distance between the left and right canine points (mm)
T*-T	Intertuberosity distance	Distance between the left and right tuberosity points (mm)
P*-P	Premaxillary width	Distance between the premaxillary points on the edge of the premaxilla (mm)
L*-P*	Alveolar cleft width	Distance between the most anterior point of the lesser segment and the nearest premaxilla point (mm)
Length I-T*T	Maxillary sagittal length	Length of the perpendicular line from the interincisive point to the intertuberosity line (mm)
Angle IMT	Premaxillary rotation	Angle between the interincisive point, the intertuberosity midpoint and the tuberosity point of the greater segment (°)
Angle C*T*T	Lesser segment rotation	Angle between the canine point of the lesser segment, the tuberosity point on the lesser segment and the tuberosity point on the greater segment (°)
Palatal size	Occlusal plane area	Projected flat area enclosed by the alveolar arch line (mm^2^)
Palatal surface area	Summed segment size	Sum of the palatal segments; greater plus lesser segment mucosa surface areas (mm^2^)
GSA	Greater segment area	Greater segment mucosa surface area enclosed by the alveolar ridge and the posterior plane comprising T and T* and being perpendicular to the plane defined by C, T, and T* (mm^2^)
LSA	Lesser segment area	Lesser segment mucosa surface area enclosed by the alveolar ridge and the posterior plane comprising T and T* and being perpendicular to the plane defined by C, T, and T* (mm^2^)

### Statistical Methods

The analysis methods were chosen for data with longitudinal measurements. Linear and nonlinear mixed-effect models (scipy and statsmodel in Python and lmer^32^ in R) were fitted with age and birth weight as fixed effects. Longitudinal data were incorporated into the model by treating patients as a random effect. Therefore, each patient may have different measurements at birth (intercept) as well as growing at a different rate (slope). Akaike Information Criterion (AIC), Bayesian Information Criterion (BIC), and log-likelihood were used to assess the models in terms of the number of parameters and prediction errors (aictab, R). The following models were evaluated: (1) age as a fixed effect with 2 random effects for each patient: a random intercept and a random slope (with respect to age); (2) age as a fixed effect with only random intercept; (3) both age and birth weight as fixed effects with random intercept and slope for each patient; (4) both age and birth weight as fixed effects with only random intercept for patients, and the effect of birth weight was tested in addition to a random effect by (5) treating age as a fixed effect with random intercept for patients and birth weight. Where linear model did not converge, nonlinear mixed-effect models (statsmodel in Python) were applied, setting age as fixed effect with random intercept for each patient, and assessed using AIC, BIC, and log-likelihood.

To generate percentile growth charts of the measurements, linear quantile mixed effect modeling (lqmm (1.5.8)^33^ in R) was used if the measurements showed linear behavior. Nonlinear percentile growth charts were generated by calculating percentile scores of residuals for each data point (scipy in Python).

Measurements were repeated for 10% of all cases, by the same rater and for 25% by a second rater. The intrarater and interrater correlation coefficients and mean absolute deviations were determined to assess the reliability of the measurements.

Simple linear regression was performed to model the relationships between the linear measurements, 2D measurements, and 3D surface measurements.

All the statistical analyses were performed using libraries for statistical tests in R 4.4.0, RStudio 2024.04.1, and Python 3.9.13.

## Results

### Patient Characteristics

Thirty patients were included in the study, and 102 models were analyzed. Table 3 shows patient characteristics. Excluding patients exhibiting Simonart's band did not change the result of the analyses; therefore, 3 such cases were included in the analyses.

**Table 3.** Characteristics of Patients Included in the Analyses (*n* = 30).

	Number patients *n* = 30
Male *n* [%]	22 (73%)
Female *n* [%]	8 (27%)
Mean birth weight in g	3506 [482]
Mean number of analyzed scans per patient	3.2 [1.2]
Number of right UCLP	16
Number of left UCLP	14
Mean age at primary surgery in month	9.13 [1.22]

Data are in number [%] or mean [standard deviation] values.

### Change in Palatal Morphology: Widths, Length, and Angles

**Figure 3.**
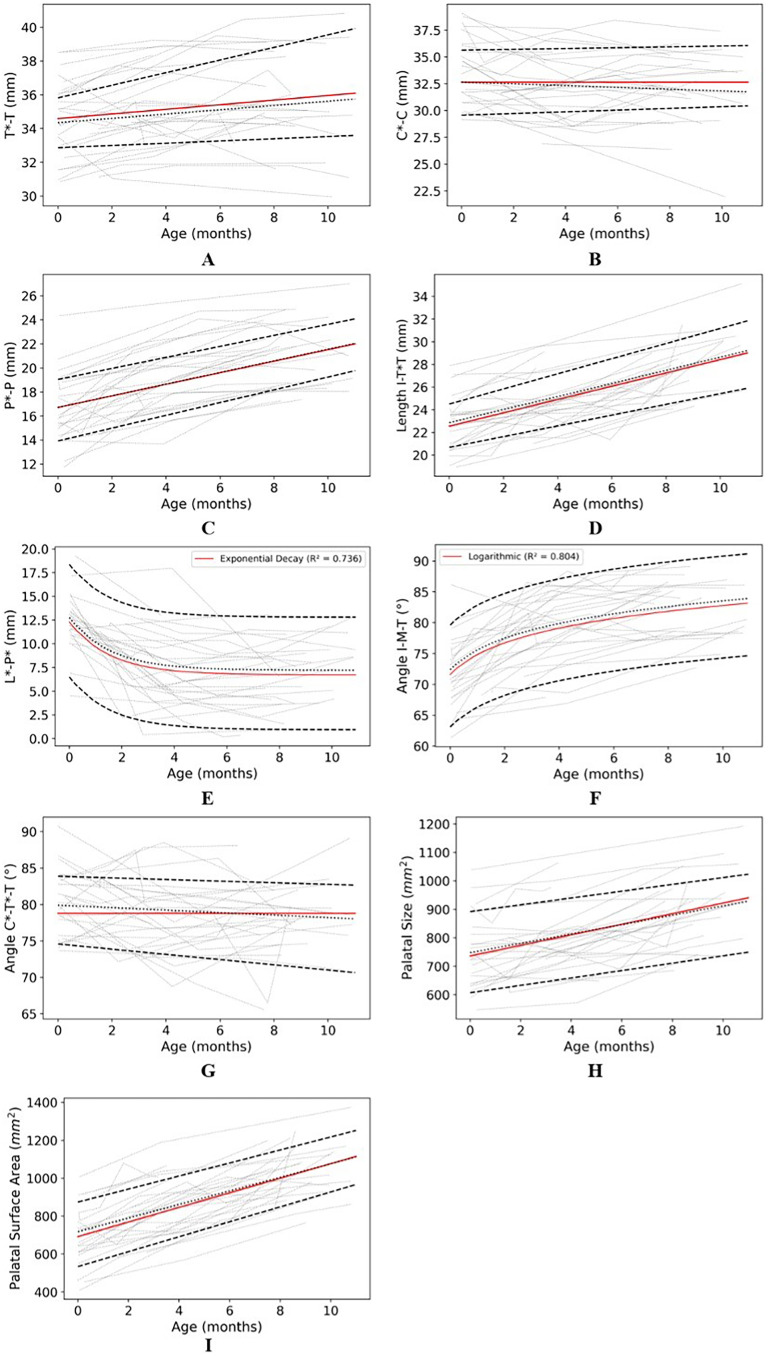
Individual Changes in the Measurements Between Birth and at the Time of Primary Surgery, Shown as Gray Dotted Lines. The red Line Shows the Best fit Curve, Linear Mixed-Effect Model, Unless Otherwise Stated. The 50th Percentiles are Shown as Black Dotted Lines and the 5th and the 95th Percentiles are Shown in Black Dashed Lines. (A) T*-T: Intertuberosity Distance, (B) C*-C: Intercanine Distance, (C) P*-P: Premaxillary Width, (D) Length I-T*T: Maxillary Sagittal Length, (E) L*-P*: Cleft Width (Exponential Decay), (F) Angle I-M-T: Premaxillary Rotation (Logarithmic), (G) Angle C*-T*-T: Lesser Segment Rotation, (H) Occlusal Plane Area, (I) Palatal Surface Area.

In this analysis, we found that adding birth weight did not improve how well the model fit the data; therefore, we only included age as the fixed effect and patients as the random effect. The results of the linear mixed-effects model and nonlinear models as appropriate for each measurement are summarized in Table S1, the growth trends, and 5th, 50th, and 95th percentile curves are shown in Figure 3. Change in palatal morphology in an example case with 3 observation time points during PSPPT up to primary surgery is presented in Figure S1.

The distances between the canines (C-C*) and tuberosities (T-T*), and the angle of rotation of the lesser segment (Angle C*-T*-T), did not change significantly until about 9 months of age. However, there was variation between patients, with some showing increases and others showing decreases. The best-fitting model suggested that the overall rate of change was minimal; however, the starting points (baseline) varied significantly between individuals. The anterior alveolar cleft width (L*-P*) decreased consistently in patients undergoing PSPPT with notable variation in the baseline. A nonlinear trend was observed, and fitting an exponential decay model improved the statistical fit, as shown in Table S1B. The angle of premaxilla rotation (Angle I-M-T) improved, approaching 90°, until approximately 9 months. The best-fitting model showed that this improvement was similar across patients. A logarithmic model was applied to account for the nonlinear trend, which further improved the model fit. Finally, the palatal surface area, projected palatal size, and premaxillary width increased at similar rates across patients, although individual baseline values varied. Adding individual variability to the rate of change did not improve model performance for all measurements, except for intercanine and intertuberosity distances.

### Growth of Palatal Surface Area

The AIC indicated that for modeling the relationship between palatal surface area and age, the linear mixed-effects model with only a random intercept (which accounts for individual patient differences in starting points) was better than the model that included both a random intercept and random slope (which would in addition account for individual differences in growth rates). In other words, the patients had different initial palatal surface areas, but their growth rates were generally similar. Including birth weight in the model, either as a fixed or random effect, did not improve the model fit. The effect of birth weight on palatal surface area and its growth rate was minimal (with a coefficient close to zero), and there was no correlation between birth weight and palatal surface area at birth or growth. The best-fitting mixed-effects model (red solid line), together with individual changes (gray dashed lines), is shown in Figure 3I, illustrating how the palatal surface area changes with age, taking into account individual patient variation. Preliminary growth chart at 5th, 50th, and at 95th percentiles (Figure 3I) show the distribution of data across different patients, including the effect of repeated measurements from the same patients.

### Intra- and Interrater Analyses

We repeated 11 scans in intra- and 23 scans in interrater analysis. Intraclass correlation coefficient based on a single rating, absolute agreement, and a 2-way mixed-effects or random-effects model, as well as mean absolute deviations between raters were calculated for each variable assessed in this study. The results are summarized in Table S2.

## Discussion

There was considerable variation in baseline measurements between patients, but the rate of change in palatal dimensions was generally consistent between them. For intercanine and intertuberosity distances, both baseline values and the rate of change varied between patients. In some cases, the width decreased as the lesser segment moved towards the greater segment, whereas in others, it increased with growth. The variation in width may be influenced by the differences in tongue pressure on the development of cleft morphology between individuals. Patients with higher tongue pressure or increased susceptibility to its effects may have a greater width at birth. Conversely, those with lower tongue pressure effects may show a reduction in width. Most patients showed minimal changes in width from birth to just prior to their primary surgery (Figure 3A and B). This may explain why the presurgical passive plate can remain in place for prolonged periods in early life without the need for frequent adjustments or replacements. This advantage is valued by centers using PSPPT, as it reduces the need for plate modifications and renewals, thereby lowering treatment burden, clinical visits, and the overall effort required from caregivers. The available tissue, represented by the sum of the palatal mucosa surface areas, increased consistently across patients, albeit from heterogeneous starting points. Similar patterns were observed for the premaxillary width, maxillary sagittal length, and projected palatal size (measured by the projected area at the occlusal plane). Apart from the palatal width and segmental symmetry, the palatal morphology, including the surface area, was comparable to that of a control group without cleft.

The growth curve indicates a linear growth pattern of the palatal surface area in the present cohort, as shown in Figure 3I. A preliminary growth chart constructed from the growth curve is also presented in Figure 3I. The percentile curves calculated in this study could be used as a reference growth chart for individual palatal morphology, to monitor palatal growth and determine the optimal timing of surgery, especially using simpler measurements, such as maxillary sagittal length, premaxillary width, or the occlusal plane area measured in a 2D photo (Figure S2). This suggests that palates with below-average surface areas are likely to remain below average over time, making catch-up growth unlikely. With a larger dataset, a more reliable growth chart could be developed, similar to those used for height, weight, and head circumference. Such reference growth charts, especially for rare conditions requiring pediatric interventions, could significantly improve treatment by providing a reference for expected growth patterns and optimizing decision making in the cleft treatment process.

### Current State and Comparison to Previous Studies

Although numerous studies have investigated the impact of interventions on UCLP morphology,^1,2,4,5,8,9,11-13,26^ our findings provide new insight into the effects of PSPPT. Previous studies, such as those by Falzoni et al,^11^ have reported significant transverse changes after cheiloplasty. Specifically, a decrease in intercanine distance and an increase in the posterior width measured as intertuberosity distance was found. However, our study showed that transverse changes are minimal during PSPPT, with limited anterior narrowing. This indicates a gradual effect of PSPPT on palatal width compared to surgical interventions.^20^ We observed consistent growth in the available tissue and projected palatal size across patients regardless of the baseline conditions. Significant variability in the absolute values of projected palatal size and morphology, as observed in this study, is consistent with the findings of Botticelli et al.^34^ This variability may be due to differences in individual effect or susceptibility to tongue pressure affecting the intertuberosity distance, as discussed above.

The impact of PSPPT on growth patterns may warrant further investigation. The linear growth curve we observed for palatal surface area suggests a predictable and stable growth trajectory, which could be valuable for long-term treatment planning. However, this observed linearity is based on a limited number of measurements, with an average of 3.2 time points per patient (Table 3). The nonlinear models reported by Bruggink et al^19^ are based on 4 measurement points in healthy palates. To allow for a meaningful comparison and to better assess the impact of PSPPT, further studies with increased measurement frequency—possibly using cross-sectional data—are needed. Growth charts based on such studies could ultimately help to monitor treatment effects, for example, between different types of PSPPT, to identify patients who may require special attention due to abnormal growth patterns, or to predict future dentofacial growth and responsiveness to dentofacial orthopedics.

### Implications and Clinical Relevance

PSPPT induces morphofunctional changes, including alterations in cleft palate anatomy, aimed at facilitating the reduction of surgical interventions toward single-stage surgical repair. However, the optimal therapy duration and timing of single-stage repair remain uncertain. On one hand, delaying single-stage repair until after 6 months of age reduces the risk of perioperative complications. On the other hand, performing the procedure before 12–18 months is preferred to minimize adverse effects on speech development. The optimal timing likely lies between these ranges and should be determined based on individual growth patterns. A case-based approach tailored to each patient's growth trajectory has the potential to yield better outcomes compared to a standardized, one-size-fits-all timeline for surgical intervention.

Data comparing growth restrictions associated with PSPPT against the individual growth potential observed in a non-PSPPT control group could provide critical insights. This comparison would enable the evaluation of PSPPT-related effects on growth and surgical outcomes.

Follow-up data can be used to create growth models in the form of growth charts to monitor palatal development, to design patient specific presurgical passive plates that incorporate projected growth, and to differentiate between intrinsic growth patterns and external intervening effects such as surgery and dentofacial orthopedics. Collinearity between measurements allows complex dimensions, such as tissue availability to be estimated from simpler metrics, such as premaxillary width and maxillary sagittal length. The percentile growth charts developed in this study could serve as a preliminary reference tool to monitor patient development and extend beyond surgical intervention. By incorporating these growth models into clinical practice, clinicians can track healthy growth rates and assess the timing and impact of interventions.

### Limitations of the Study and Further Research

The small sample size in this study limited the analysis of covariates and prevented the creation of highly reliable growth charts. Larger standardized datasets are needed for future research and clinical implementation. In addition, the results have limited applicability to palatal development under other types of PSPPT. Future studies should include the generation of growth charts for untreated patients with UCLP and patients without cleft palate to delineate the effects of PSPPT and the intrinsic developmental pattern in the palate with cleft. Clinically, growth charts based on simpler linear measurements—such as premaxillary width, maxillary sagittal length, or projected palatal size—may provide a more practical approach to increase sample sizes for growth charts and tracking palatal growth in clinical routine. In the future, automated measurement tools applied to digital palatal impressions will improve measurement accuracy and facilitate their integration into routine clinical practice.

## Conclusions

Although there was a large variation in baseline palatal size between patients, the rate of growth in all dimensions was similar across individuals. Therefore, growth charts for palatal morphology can be created to monitor the morphological changes prior to surgical repair. They could be used as a reference to assess individual intrinsic development and to predict future growth in response to treatment. These growth charts have the potential to provide objective reference values, allowing treatment effects to be included as additional factors in growth assessments.

## Supplemental Material

sj-docx-1-cpc-10.1177_10556656251369672 - Supplemental material for Development of Palatal Growth Charts for Unilateral Cleft Lip and Palate Before Primary Single-Stage Repair Supplemental material, sj-docx-1-cpc-10.1177_10556656251369672 for Development of Palatal Growth Charts for Unilateral Cleft Lip and Palate Before Primary Single-Stage Repair by Adelita Sommacal, Benito K. Benitez, Yoriko Lill, Maria Fernandez-Pose, Michel Beyer and Florian M. Thieringer, Andreas A. Mueller in The Cleft Palate Craniofacial Journal

sj-docx-2-cpc-10.1177_10556656251369672 - Supplemental material for Development of Palatal Growth Charts for Unilateral Cleft Lip and Palate Before Primary Single-Stage Repair Supplemental material, sj-docx-2-cpc-10.1177_10556656251369672 for Development of Palatal Growth Charts for Unilateral Cleft Lip and Palate Before Primary Single-Stage Repair by Adelita Sommacal, Benito K. Benitez, Yoriko Lill, Maria Fernandez-Pose, Michel Beyer and Florian M. Thieringer, Andreas A. Mueller in The Cleft Palate Craniofacial Journal
