# Supplementary material for: Development of Palatal Growth Charts for Unilateral Cleft Lip and Palate Before Primary Single-Stage Repair
Source: Cleft Palate Craniofac J. 2025 Aug 26;63(7):2203–11. doi: 10.1177/10556656251369672 (PMC13260759; doi:10.1177/10556656251369672)
Supplement: sj-docx-1-cpc-10.1177_10556656251369672 - Supplemental material for Development of Palatal Growth Charts for Unilateral Cleft Lip and Palate Before Primary Single-Stage Repair [file sj-docx-1-cpc-10.1177_10556656251369672.docx]

**Supplemental Material**

**Table S1.**

**(A)** Each measurement was fitted to models (1) ~ Age + (Age|Patient) (2) ~ Age + (1|Patient) (3) ~ 1 + (Age|Patient) (4) ~ 1 + (1|Patient). Results of the model with the best fit are presented.

| **Measurement** | **Formula and description** | **Fixed Effect** | | | | | **Random Effect** | | **Model Statistics** | | |
| --- | --- | --- | --- | --- | --- | --- | --- | --- | --- | --- | --- |
|  |  | **Fixed Effect** | **Estimate** | **Std. Error** | **95% CI Lower** | **95% CI Upper** | **Random Effect** | **Std. Dev.** | **AIC** | **BIC** | **Log Likelihood** |
| **T*-T**  **(intertub. dist.)** | T*-T ~ Age + (Age\|Patient)  Interuberosity distance | (Intercept) | 34.59*** | 0.40 | 33.79 | 35.39 | Intercept (Patient) | 2.06 | 378.35 | 393.80 | -183.17 |
|  |  | Age | 0.14** | 0.05 | 0.04 | 0.23 | Slope (Age\|Patient) | 0.22 |  |  |  |
| **C*-C**  **(intercanine dist.)** | C*-C ~ 1 + (Age\|Patient)  Intercanine distance | (Intercept) | 32.65*** | 0.50 | 31.60 | 33.70 | Intercept (Patient) | 2.74 | 450.14 | 463.07 | -220.07 |
|  |  |  |  |  |  |  | Slope (Age\|Patient) | 0.33 |  |  |  |
| **P*-P**  **(premaxil. width)** | P*-P ~ Age + (1\|Patient)  Premaxillary width | (Intercept) | 16.72*** | 0.49 | 15.75 | 17.69 | Intercept (Patient) | 2.43 | 412.11 | 422.45 | -202.05 |
|  |  | Age | 0.48*** | 0.04 | 0.41 | 0.55 |  |  |  |  |  |
| **Length I-T*T**  **(maxil. length)** | Length ~ Age + (1\|Patient)  Maxillary sagittal length | (Intercept) | 22.55*** | 0.43 | 21.70 | 23.41 | Intercept (Patient) | 2.00 | 412.82 | 423.12 | -202.41 |
|  |  | Age | 0.59*** | 0.04 | 0.50 | 0.67 |  |  |  |  |  |
| **Angle C*-T*-T**  **(premaxil. rotation)** | Angle C*T*T ~ 1 + (1\|Patient)  Lesser segment rotation | (Intercept) | 78.78*** | 0.71 | 77.36 | 80.18 | Intercept (Patient) | 3.20 | 574.26 | 581.99 | -284.13 |
| **Palate Size at Occlusal Plane** | Palate Size ~ Age + (1\|Patient) | (Intercept) | 736.33*** | 22.35 | 691.97 | 780.44 | Intercept (Patient) | 109.53 | 1113.44 | 1123.65 | -552.72 |
|  |  | Age | 18.57*** | 1.80 | 14.99 | 22.10 |  |  |  |  |  |
| **Palatal Surface Area** | Surface Area ~ Age + (1\|Patient)  Summed segment area | (Intercept) | 691.92*** | 26.05 | 640.22 | 743.05 | Intercept (Patient) | 114.75 | 1175.95 | 1186.17 | -583.97 |
|  |  | Age | 38.50*** | 2.79 | 32.91 | 43.96 |  |  |  |  |  |

*** p < 0.001; ** p < 0.01; * p < 0.05

**(B)** Nonlinear models for measurements which showed deviation from linear behavior; exponential decay for the alveolar cleft width (L*-P*) and logarithmic model for the premaxillary rotation (Angle I-M-T) was fitted.

| **Measurement** | **Model** | **Formula** | **Parameters [a, b, c]** | **AIC** | **BIC** | **Log-Likelihood** | **R-squared** |
| --- | --- | --- | --- | --- | --- | --- | --- |
| **L*-P*** | Exponential Decay | L*-P* ~ **a**·exp(-**b**·Age) + **c** + (1\|Patient) | [5.56, 0.64, 6.72] | 497.16 | 504.85 | -245.58 | 0.74 |
| **Angle I-M-T** | Logarithmic | Angle IMT ~ **a**·log(Age + 1) + **b** + (1 \| Patient) | [4.66, 71.61] | 572.91 | 578.06 | -284.45 | 0.80 |

**Table S2:** Intra- and inter-rater analyses by ICC and mean absolute deviations between the raters.

| **Measurement** | **Intra-rater** | | | | **Inter-rater** | | | |
| --- | --- | --- | --- | --- | --- | --- | --- | --- |
|  | **ICC** | **ICC 95% CI** | **Mean Deviation** | **n** | **ICC** | **ICC 95% CI** | **Mean Deviation** | **N** |
| **T*-T** | 0.91 | [0.71, 0.98] | 0.77 | 11 | 0.83 | [0.65, 0.93] | 1.24 | 23 |
| **C*-C** | 0.99 | [0.98, 1.00] | 0.19 | 11 | 0.97 | [0.92, 0.99] | 0.56 | 23 |
| **P*-P** | 0.98 | [0.94, 1.00] | 0.27 | 11 | 0.83 | [0.64, 0.92] | 1.02 | 23 |
| **L*-P*** | 0.99 | [0.98, 1.00] | 0.22 | 11 | 0.92 | [0.78, 0.97] | 0.86 | 19 |
| **Length** | 0.89 | [0.64, 0.97] | 1.10 | 11 | 0.91 | [0.80, 0.96] | 0.85 | 23 |
| **Angle I-M-T** | 0.94 | [0.78, 0.98] | 0.79 | 11 | 0.95 | [0.88, 0.98] | 1.52 | 23 |
| **Angle C*-T*-T** | 0.93 | [0.75, 0.98] | 1.90 | 11 | 0.81 | [0.61, 0.92] | 2.86 | 23 |
| **Palatal Size** | 0.90 | [0.68, 0.97] | 44.95 | 11 | 0.90 | [0.79, 0.96] | 37.51 | 23 |
| **Palatal Surface Area** | 0.96 | [0.86, 0.99] | 46.07 | 11 | 0.93 | [0.83, 0.97] | 45.90 | 21 |


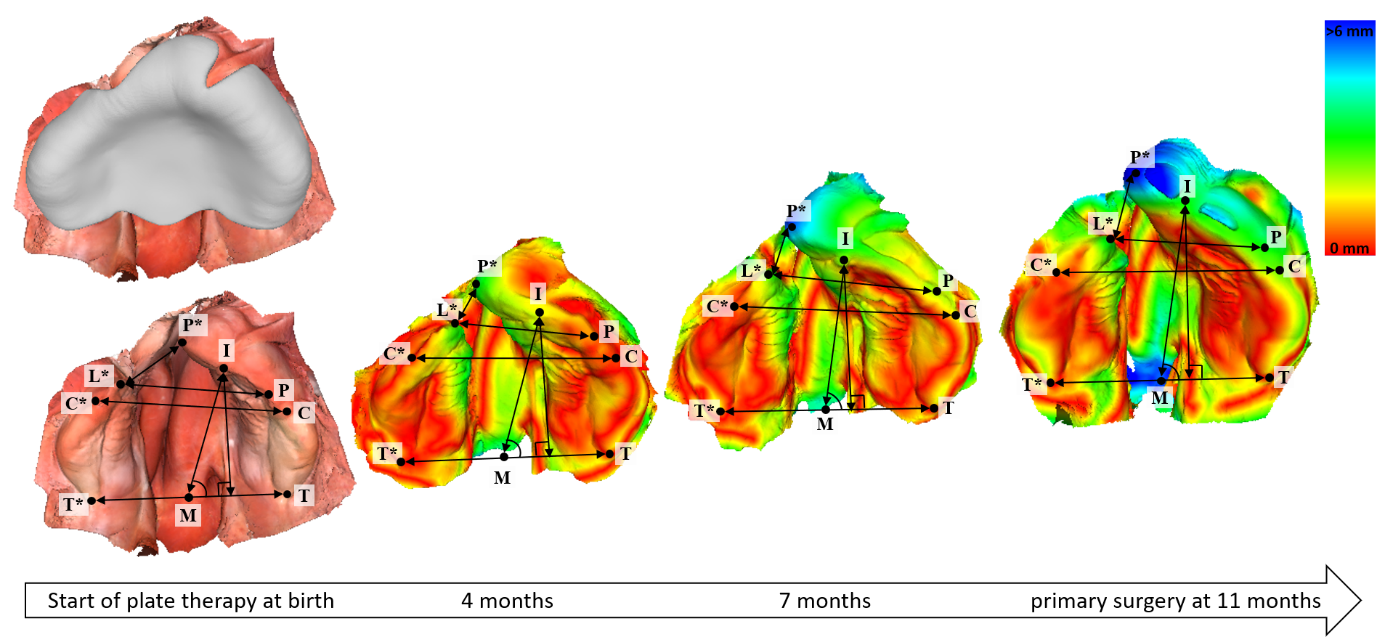
**Figure S1.** An Example demonstrating intraoral scans acquired at birth for an individualized plate design, followed by scans at approximately 4 and 7 months to monitor plate therapy, and at around 11 months just before the primary one-stage surgical closure of cleft lip and palate. Heatmap shows the Hausdorff distances from the scan at birth. The linear measurements analyzed in this study are depicted.


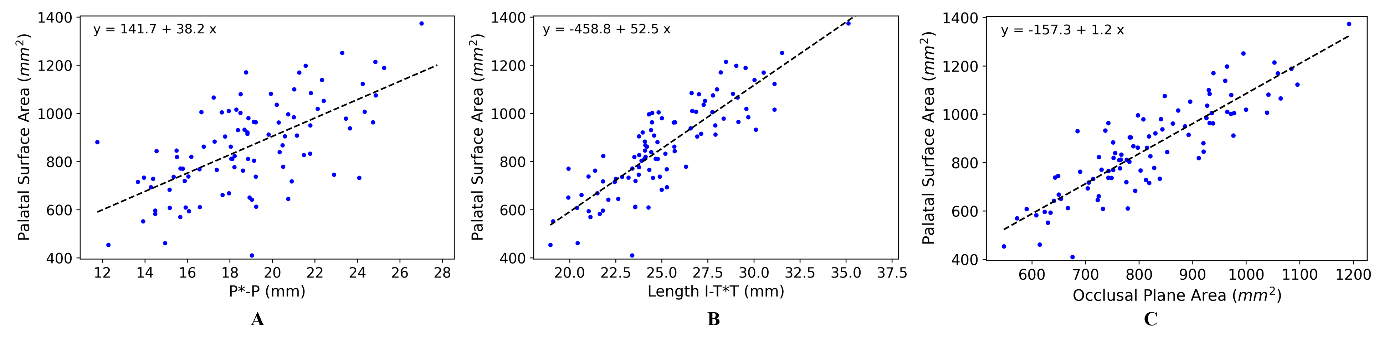


**Figure S2.** Estimation of palatal surface area (greater + lesser segment areas) based on linear measurements, (A) P*-P: premaxillary width (B) Length I-T*T: maxillary sagittal length defined as the length of the perpendicular line from the interincisive points to the intertuberosity line (C) Occlusal plane area representing the palatal size measured in two dimensions (2D) projected onto occlusal plane.
